# Automated reconstruction of whole-embryo cell lineages by learning from sparse annotations

**DOI:** 10.1038/s41587-022-01427-7

**Published:** 2022-09-05

**Authors:** Caroline Malin-Mayor, Peter Hirsch, Leo Guignard, Katie McDole, Yinan Wan, William C. Lemon, Dagmar Kainmueller, Philipp J. Keller, Stephan Preibisch, Jan Funke

**Affiliations:** 1 HHMI Janelia, Ashburn, VA, USA; 2 Max Delbrück Center for Molecular Medicine in the Helmholtz Association, Berlin, Germany; 3 Faculty of Mathematics and Natural Sciences, Humboldt-Universität zu Berlin, Berlin, Germany; 4 CNRS, UTLN, LIS 7020, Turing Centre for Living Systems, Aix Marseille University, Marseille, France; 5 MRC Laboratory of Molecular Biology, Cambridge, UK; 6 Biozentrum, University of Basel, Basel, Switzerland

## Abstract

We present a method to automatically identify and track nuclei in time-lapse microscopy recordings of entire developing embryos. The method combines deep learning and global optimization. On a mouse dataset, it reconstructs 75.8% of cell lineages spanning 1 h, as compared to 31.8% for the competing method. Our approach improves understanding of where and when cell fate decisions are made in developing embryos, tissues, and organs.

With recent advances in light-sheet imaging techniques, it is possible to acquire whole embryo developmental datasets over long time scales with high spatial and temporal resolution in complex organisms such as mouse, *Drosophila*, and zebrafish^1^. The resulting datasets contain information required to track the movement and division of nuclei over time, yielding lineage trees and quantitative data on cellular dynamics that are crucial to the study of developmental biology at the cellular level^2^. However, manually tracing lineages with dedicated tools like MaMuT^3^ or Mastodon (https://github.com/mastodon-sc/mastodon) is arduous, and for complex, developing organisms it is only feasible to annotate a small percentage of all tracks, making automatic cell tracking necessary for holistic analysis.

Cell-tracking algorithms have been developed for and tested on diverse datasets with different characteristics. While hand-engineered features are sufficient for cell detection and tracking in some model organisms^4,5^, learned dataset-specific features, given sufficient training data, improve performance for datasets with heterogeneous cell or nucleus phenotypes and varying imaging statistics over time and space. In particular, deep learning has been shown to improve cell detection^6,7^, segmentation^8–11^, and tracking^7,11–14^ on a variety of datasets. Additionally, it has been shown that tracking methods that take into account global spatiotemporal context perform better, especially for datasets with more movement between time frames^13^. Tracking by graph optimization over a large spatiotemporal context allows inclusion of biological knowledge about track length and cell cycle, improving track continuity^6,15,16^and even allows recovery from noisy detection and segmentation^17^.

Only a few of the aforementioned cell-tracking methods are readily applicable to the unique challenges posed by contemporary four-dimensional (3D+t) light-sheet datasets, the focus of this work. Practical methods for this kind of data should take into account temporal and 3D spatial context, easily scale to multi-terabyte datasets, and ideally should not require a manual segmentation of cells for training, owing to the time required to generate per-pixel ground truth. Of methods that fulfill these requirements, tracking with Gaussian mixture models (TGMM)^5^ has been shown to work well on model organisms with approximately ellipsoid nuclei. More recently, the ELEPHANT tracking platform employed deep learning for cell detection and per-frame linking in light-sheet datasets with diverse cell appearance and movement^12^. ELEPHANT requires a manual pseudo-segmentation of nuclei by ellipsoid fitting, which takes less time to generate than a per-pixel manual segmentation, but more than point annotations.

Our method combines global optimization and learned features, generating cell lineages through global graph optimization with learned costs. We show that this combination substantially decreases tracking error on three diverse datasets of different model organisms with different temporal resolution, signal-to-noise ratio, and nuclear appearance. Features are learned from sparse point annotations produced by current manual lineage tracking tools like MaMuT and Mastodon, and thus do not require a manual segmentation or dense lineage annotations, which allows rapid generation of training data. Crucially, the steps of our method—including the global optimization—can be computed in a distributed fashion, which is necessary to process multi-terabyte light-sheet datasets and enable the study of whole embryo morphogenesis.

An overview of our method is shown in Fig. 1. Because we are learning features from the data, the method is not tied to a specific input type or format: we use fused and unfused light-sheet recordings with a single fluorescent nuclear channel, and could easily extend to multi-channel input. We use sparse point annotations to train a convolutional neural network to predict at each pixel a ‘cell indicator’ value that peaks at the center of each nucleus^6,18^. In contrast to ref. ^6^ we additionally predict a ‘movement vector’ that points to the center of the same cell nucleus in the previous time frame^7,12^. From these predictions, we generate a candidate graph in two steps: first, we place nodes at the local maxima of the cell indicator values to represent possible cell center locations, with a score to encode the confidence of the network; and second, we locally connect nodes in adjacent frames with edges to represent the possibility that the nodes represent the same cell, and assign a score to each edge on the basis of agreement with the predicted movement vector.

Next, we solve a globally constrained optimization problem on the candidate graph to select a subset of nodes and edges that form coherent lineage trees. We know that between time frames, cells can move, divide into two, enter or leave the field of view, or die, but not merge or split into more than two. Thus, we introduce tree constraints to prevent merging and divisions producing more than two progeny. The objective function incorporates prior knowledge that cell movement is much more common than division or death. We further encourage long and continuous lineages by penalizing the start of new tracks. These tree constraints and continuity costs are similar to those in previous work^6,15,17^; however, we also incorporate the node and edge scores generated by the neural networks into the objective function as learned costs. Thus, we optimize for valid lineages that are both continuous and supported by the learned cell location and movement features. While ref. ^6^ employ a minimum-cost flow solver on the whole dataset, our integer linear program (ILP) formulation of the optimization problem additionally allows block-wise solving in parallel on large datasets by introducing additional constraints to ensure consistent solutions between adjacent blocks.

We evaluate our method on three sparsely annotated datasets from different commonly used model organisms to study embryogenesis: mouse ^19^, *Drosophila*^5^, and zebrafish ^20^ (see Supplementary Note 1 for details about the datasets and annotations). We compare the performance of our method against TGMM, the previous state-of-the-art method on these datasets^5,19^, and greedy tracking using a per-frame nearest neighbor linking algorithm similar to the ELEPHANT tracking method^12^. We compute multiple metrics, including the fraction of perfectly constructed lineages over a range of time periods, and errors per ground truth edge, broken into the following error types: false negative edges (FN), identity switches (IS; when two tracks switch off following the same cell), false positive divisions (FP-D), and false negative divisions (FN-D) (Supplementary Figure 1). False positive edges cannot be computed using sparse ground truth, because we cannot tell if unmatched reconstructions are false positives or tracking unannotated cells, and thus they are not included in our quantitative analysis. We show in Fig. 2 that, with around 20 hours of ground-truth annotation effort, our method correctly reconstructs more cell lineages than both baselines over all time ranges for all datasets. The largest improvement over TGMM is on the mouse dataset: our method correctly tracks 75.8% of mouse cells over a time span of 1 h (12 time frames), compared with 31.8% for TGMM. By 175 min (35 frames), our method still correctly tracks more than half of all cells, while TGMM tracks less than 8%. On all three datasets, our method greatly reduces false negative edges as compared to TGMM, while when compared with the greedy baseline, our method produces far fewer false positive divisions. Supplementary Note 2 contains a detailed description of the evaluation metrics and baselines, and further observations about the performance on various metrics across organisms and evaluation regions.

Both the candidate graph generation and lineage optimization steps of our method are fully parallelizable and scale linearly with the size of the recording, which enables dense lineage reconstruction on very large datasets in reasonable time. On 20 GPUs and 100 CPU cores, reconstruction of dense lineages took about 44 h on the 4.7 TB mouse dataset, generating more than 7 million cell detections and 360,000 tracks over the 44-h recording. Given the estimates that there are 6 million true cell detections in the dataset and that an annotator can click on a cell center every 1.5−3 s, it would take 2,500−5,000 annotator-hours to manually trace all lineages in this dataset. The source code of our method is publicly available, together with training and inference scripts and extensive documentation (https://github.com/funkelab/linajea).

The ability to densely reconstruct cell lineages in such large, information-rich datasets opens up vast opportunities for exploring cell fate dynamics and tissue morphogenesis. Accurately following cells and their progeny over extended time periods allows identification of individual cell behaviors that are not visible with shorter and less accurate lineages. For example, being able to accurately track more than half of all cells over a time window of 175 min in the mouse dataset, as compared to only 30 min with previous methods, greatly reduces the manual curation needed to test hypotheses such as the existence of neuromesodermal progenitors that can produce neural or mesodermal progeny. While further work is required to improve detection of cell division, the dense cell lineages we publish with this method are a rich source of information about the development and cell fate dynamics of common model organisms.

## Method

### Network architecture, training, and prediction

To attain per-voxel predictions for cell locations and movements, we use a U-Net architecture with four resolution levels^21^. Our choice of the U-Net architecture over alternatives like Mask-RCNN or YOLO is motivated by two points: first, we found that the performance of the U-Net for cell detection is sufficient for our use case (in particular, regarding high recall for subsequent filtering through the ILP); and second, our proposed movement vectors require dense predictions, which can be naturally generated with a U-Net architecture. We also chose the same architecture for the cell indicators to unify, and thus simplify, our pipeline. To incorporate temporal as well as spatial context, we concurrently feed seven 3D frames centered on the target time point and use four-dimensional convolutions until, owing to valid convolutions, the time dimension is reduced to 1. We downsample by factors (2, 2, 2) in xyz, except for anisotropic datasets (mouse, *Drosophila*) where we do not downsample in *z* in the first downsampling layer. We use 12 initial feature maps and increase by a factor of 3 at each level. When upsampling, we restrict our upsampling convolutional kernels to constant values, as we have observed this reduces artifacts in the output.

The cell indicator network is trained on sparse point annotations and predicts the centers of cell nuclei. The training signal for this network, called the cell indicator value, is a Gaussian with maximum value 1 at the cell center annotation and decreasing according to a hyperparameter *σ*. With only sparse annotations, it is unknown if pixels far from cell center annotations are background or cells that were not annotated. To avoid training on unknown regions, we construct a ‘training mask’ around each annotation with a user-defined radius. This radius should be small enough that the mask will not overlap with neighboring cell nuclei. We only train on the mean squared error loss within the training mask. We are not training our cell indicator network on any background regions, so the behavior is unconstrained in the background. After prediction, we use local non-maximum suppression (NMS) to extract cell center candidates, with the goal of detecting all cell centers along with potential false positives owing to the unconstrained background behavior. The NMS window size is dataset dependent and should be a bit smaller than the minimal distance between two cell centers. To reduce the number of false positives, especially in background regions, we only consider detections above a threshold cell indicator value, determined empirically for each dataset and model. Additionally, if a foreground mask is available (as in the zebrafish dataset) we filter detections to those that lie in the foreground.

In addition to the cell indicator network, we train a movement vector network to predict the movement of cells between frames. For a pixel near to a cell in frame *t*, the movement vector is a 3D offset vector that points to the relative location of the center of the same cell in frame *t* — 1. Predicting the offset to the same cell in the previous time frame, rather than the next time frame, allows divisions to be represented naturally, since each daughter cell points to the center of the parent cell. We calculate the loss on two different masked regions. Loss *L*_A_ is the mean squared error between the ground truth and predicted movement vectors, calculated over the same training mask as the cell indicator network. Loss *L*_B_ limits the error to voxels with maximal cell indicator values after NMS that also are within the training mask. The total loss is the weighted sum *L* = α*L*_A_(1-α)*L*_B_ with $\alpha =\frac{1}{1+\exp\;0.01\;(-i+20,000))}$ and *i* being the number of training iterations. This weighting scheme weights *L*_A_ higher at the beginning of training, when the cell indicator network is still converging, with a smooth transition to *L*_B_ at 20,000 iterations.

These networks are trained simultaneously for 400,000 iterations, with batch size 1. Batches are randomly sampled from annotated locations, and random augmentations including elastic deformation, mirroring, transposing axes, and intensity augmentation are applied using the GUNPOWDER library (https://github.com/funkey/gunpowder). Prediction is then performed block-wise using parallelization over multiple GPUs to process large datasets efficiently. To eliminate edge artifacts, we ensure that our prediction stride is a multiple of the network downsample factors^22^.

### Candidate graph extraction

After prediction, we create a directed candidate graph *G* = (*V, E*) with nodes that represent possible cell center locations and edges that represent possible movements of the same cell between frames. *G* is expected to contain extra nodes and edges, which will be filtered out in the final step.

*V* is the set of NMS detections. Each *v* ∈ *V* has a three-dimensional location *l_v_*, a time *t_v_*, a predicted cell indicator score *s_v_*, and a predicted movement vector **m**_*v*_. We avoid storing the predicted cell indicators and movement vectors at every pixel by performing NMS on the cell indicator values during prediction and only save the predicted values at the detection.

We construct the set of directed edges *E* by locally connecting nodes in adjacent frames with edges that point one frame backwards in time. For each candidate *v* at time *t_v_*, we compute the predicted location *l_v_* of the same cell in the previous frame: ${\hat{l}}_{v}=l_{v}+m_{v}$ Then, we add an edge from *v* to each node candidate *u* at time *t_v_* — 1 where the predicted distance ${\hat{d}}_{e}\;=||\;{\hat{l}}_{v}-l_{u}|{|}^{2}$ is less than a hyperparameter *θ*. ${\hat{d}}_{e}$ is stored as a score on each edge.

### Discrete optimization to find lineage trees

We construct a lineage tree by selecting a subset of nodes and edges from *G*. We define a vector **y** = [**y**^*V*^, **y**^*E*^]**^T^** ∈ {0, 1}^|*V*|+|*E*|^ such that each element of the vector corresponds to a node or edge in *G*. Then *G*(**y**) is the sub-graph induced by **y**that only contains nodes and edges with the corresponding element of **y** equal to 1.

We then construct a constrained optimization problem that minimizes the objective

$$\underset{\text{y}}{\min}\;C\left(\text{y}\right)\;\text{s}.\text{t}.\;G\left(\text{y}\right)\;\in \;{𝒯}^{2}$$,

where 𝒯^2^ is the set of binary forests and *C* : **y** → ℝ assigns a cost for each set of selected nodes and edges. Thus, the goal is to select the cost-minimal subset of nodes and edges from *G* that form a binary forest.

To simplify the presentation of the cost function, we introduce two auxiliary indicator vectors of length |*V*| that can be entirely derived from **y**. The indicator for tracks appearing, **y**^*T*^, is 1 for nodes at the beginning of a track and 0 otherwise. **y**^*D*^ represents a division and is 1 for the parent node of a division. For a formalization of the definition of these auxiliary vectors from **y**, see below.

With these auxiliary indicator variables, we define a linear cost function as follows:

(1) $$C\left(\text{y}\right)=\langle \text{c},\;\text{y}\rangle +\langle {\text{c}}^{T},\;{\text{y}}^{T}\rangle +\langle {\text{c}}^{D},\;{\text{y}}^{D}\rangle$$,

where **c** = [**c**^*V*^, **c**^*E*^]^**T**^ is a vector containing the cost for selecting each node and edge, **c**^*T*^ is a vector containing the cost *c^T^* of having to start a new track and **c**^*D*^ is a vector containing the cost *c^D^* of having a division occurring. The costs *c^T^* and *c^D^* are constant parameters of the method, but the predicted cell indicator values and movement vectors are used to individualize the cost vector **c** for selecting each node and edge.

With *s_i_* as the cell indicator score for node *i*, we define the node selection cost for node *i* as $c_{i}^{V}=\tau^{V}+w^{V}s_{i}$ where *τ^V^* and *w^V^* are parameters of the method. To encourage selection of higher cell indicator scores during minimization, *w^V^* should be negative.

Similarly, with ${\hat{d}}_{i}$ as the distance between the predicted and actual offsets at edge *i*, we define the edge selection cost for edge *i* as $c_{i}^{E}=w^{E}{\hat{d}}_{i}$. Unlike with node scores, *w^E^* should be positive to encourage selection of edges with lower scores, since those edges align better with the predicted cell movement.

To determine the optimal values of the ILP parameters *c^T^*, *c^D^*, *τ^V^*, *w^V^*, and *w^E^*, we performed a grid search where we fixed *c^D^* = 1 to eliminate redundant solutions. We selected the parameter set that minimized the sum of errors over the validation set (Supplementary Note 2).

*Integer linear program formulation*. We use an ILP to solve the constrained optimization problem with the Gurobi solver (https://www.gurobi.com/). The objective is the cost function *C*(**y**) (Eq.1). To ensure a binary forest with correctly set auxiliary variables, we implement three kinds of constraints: consistency, continuation, and split constraints.

The first consistency constraint requires that if an edge is selected, the incident nodes are selected as well. This constraint for edge *e* = (*v*,*u*) is represented by the equation *2y_e_*—*y_v_*—*y_u_*≤ 0. The second consistency constraint ensures that the number of selected incoming edges is ≤2, that is, for each node *v*: $\sum_{n\in N_{v}}y_{n}-2y_{v}\leq 0$ with *N_v_* being the set of edges from nodes in *t_v_* + 1 to *v*. The continuation constraint ensures that if a node is selected either it is marked as the beginning of a track or the track continues. Let *P_v_* be the set of edges from node *v* in time *t_v_* to nodes in *t_v_* — 1. We define the track continuation constraint as $\sum_{p\in p_{v}}y_{p}+y_{v}^{T}-y_{v}=0$ ensuring that if node *v* is selected, either there is exactly one selected edge to time *t_v_* — 1 or the track (appear) indicator $y_{v}^{T}$ is set to 1 (and thus the associated cost has to be paid). Finally, the two split constraints ensure that the division indicator $y_{v}^{D}$ is set for every cell that divides, that is, for each node $v:\;\sum_{n\in N_{v}}y_{n}-y_{v}^{D}\leq 1$ and $\sum_{n\in N_{v}}y_{n}-2y_{v}^{D}\geq 0$

*Processing large volumes block-wise*. Ideally, we would solve the ILP for the whole candidate graph at once to obtain a globally optimal solution. However, for large volumes, this is too time and memory intensive. Therefore, to obtain lineage trees for arbitrarily large volumes, we divide the candidate graph into a set of blocks *B* that tile the whole volume and use multiple processes to solve the ILP for many blocks in parallel.

Solving each block *b* ∈ *B* completely independently can result in discontinuities in tracks between blocks, and the constraints would no longer be fulfilled at the boundaries. To ensure a consistent, valid solution across the whole volume, we allow each process to view a context region around the target region *b* that must be at least as large as the amount a cell can move in space between two frames and contain at least one time frame in each direction. Let $\hat{b}$ be the union of *b* and the surrounding context area. A process reads all nodes and edges in $\hat{b}$, solves the ILP, and writes the result for only the target region *b* into a central database. If the database already contains results in the context region, these selections will be introduced as further constraints into the ILP, ensuring the solution will be consistent across boundaries. At the block boundaries, we set the new track cost c^T^ to zero, because we do not want to penalize solutions that cross block boundaries.

The introduction of a context region introduces dependencies between neighboring blocks, and thus they cannot be run trivially in parallel. By ensuring that overlapping blocks are never run simultaneously using the DAISY library (https://github.com/funkelab/daisy), we obtain a globally consistent solution while retaining a high degree of parallel processing. While there is no guarantee of global optimality, with a large enough context region, we assume that nodes and edges further away do not affect the local solution in a target region.

The number of frames in a block influences the choice of optimal ILP hyperparameters. Therefore the size used in the grid search has to be the same, or at least of the same magnitude, as during inference.

## Supplementary Material

Supplementary data

Supplementary information

## Figures and Tables

**Fig. 1 |:**
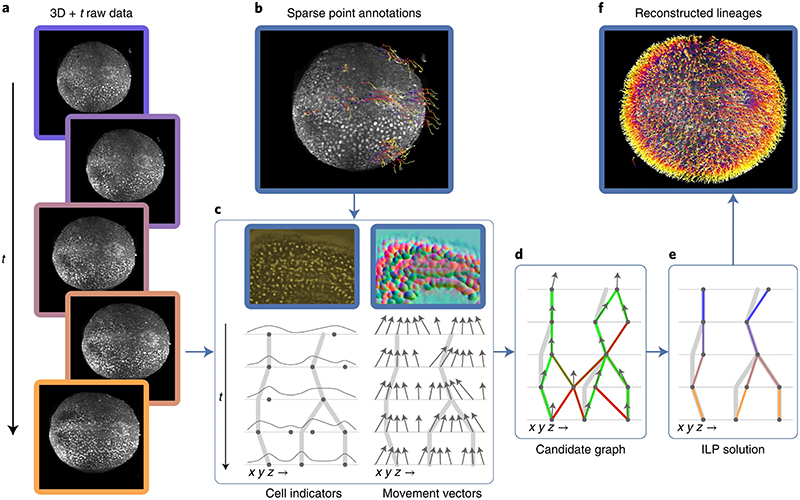
Overview of the method, including data and results from the mouse dataset. **a**, Raw mouse data over 50 time points, visualized as a max intensity projection. **b**, Sparse point annotations superimposed over the first frame of the raw data. Purple dots show the locations of annotated cells in the first time point, and the tails show the movement over time. **c**, Top, examples of the cell indicators (shown as intensity values) and 3D movement vectors (shown as RGB). The cell indicator is trained to have maxima at the center of each nucleus, and the movement vector network is trained to predict the relative location of the same cell nucleus in the previous time point. Bottom, sketch of the predictions over time. Thick gray lines represent ground truth lineages, dots are cell indicator maxima, and arrows are movement vectors. **d**, Candidate graph extracted from the network output. Candidate cells are at cell indicator maxima, and nearby cells are connected with edges that are scored by agreement with the movement vector. **e**, Consistent lineage trees extracted from the candidate graph by global optimization using learned features and biological priors. **f**, Densely reconstructed lineages visualized over the mouse data.

**Fig. 2 |:**
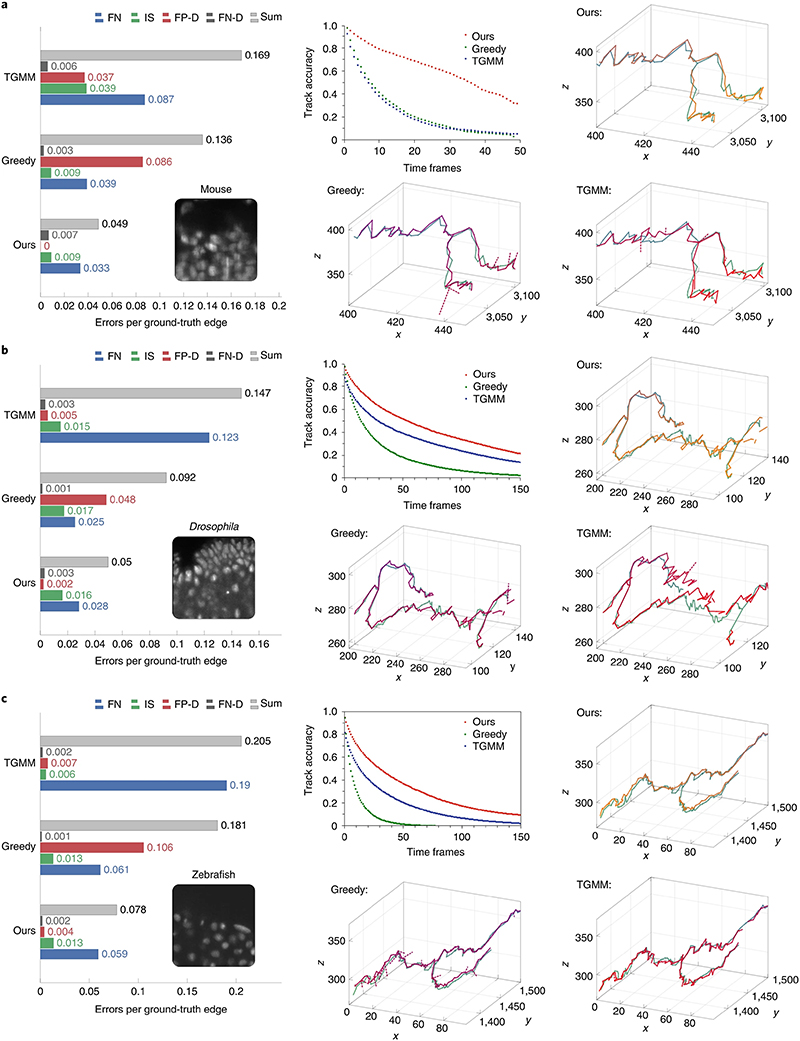
Comparison of tracking errors on three datasets. **a−c**, Comparison of tracking errors on mouse (**a**), *Drosophila* (**b**), and zebrafish (**c**) datasets. **a−c**, Left, average errors per ground truth edge for each error type; middle top, fraction of error-free tracks for a given track length; middle bottom, top right, and bottom right, example ground truth track (green) with superimposed tracking result (orange or red) for our method (top right), the greedy baseline (middle bottom), and TGMM (bottom right), respectively. Other than the dashed false positive divisions, we only show detections that matched the selected ground truth track.

## Reporting summary

Further information on research design is available in the Nature Research Reporting Summary linked to this article.
