# Supplementary material for: Automated reconstruction of whole-embryo cell lineages by learning from sparse annotations
Source: Nat Biotechnol. 2022 Sep 5;41(1):44–9. doi: 10.1038/s41587-022-01427-7 (PMC7614077; doi:10.1038/s41587-022-01427-7)
Supplement: Supplementary file 1 — Supplementary Notes 1–4, Supplementary Discussion, Supplementary Figs 1–7, Supplementary Tables 1–5. [file 41587_2022_1427_MOESM1_ESM.pdf]

nature portfolio

https://doi.org/10.1038/s41587-022-01427-7

Supplementary information

Automated reconstruction of whole-embryo cell lineages by learning from sparse annotations

In the format provided by the authors and unedited

Automated Reconstruction of Whole-Embryo Cell Lineages by Learning from Sparse Annotations

Supplemental Material

Caroline Malin-Mayor[1], Peter Hirsch[2,3], Leo Guignard[1,4], Katie McDole[1,5], Yinan Wan[1,6], William C. Lemon[1], Dagmar Kainmueller[2,3], Philipp J. Keller[1], Stephan Preibisch[1], Jan Funke[1]

1: HHMI Janelia, Ashburn, USA
2: Max Delbrück Center for Molecular Medicine in the Helmholtz Association, Berlin, DE
3: Humboldt-Universität zu Berlin, Faculty of Mathematics and Natural Sciences, Berlin, DE
4: Aix Marseille Univ, CNRS, UTLN, LIS 7020, Turing Centre for Living Systems, Marseille, FR
5: MRC Laboratory of Molecular Biology, Cambridge, UK
6: Biozentrum, University of Basel, Basel, CH

Supplementary Note 1: Datasets and Annotations

We test our cell tracking method on time-lapse light-sheet recordings from three common model organisms: *Drosophila*, mouse, and zebrafish. We call these datasets Droso, Mouse, and ZFish, respectively, and summarize some relevant information about them in Supplementary Table 1. Each dataset records a fluorescent nuclear marker: for ease of discussion, we will refer to each nucleus as corresponding to a single cell. While two of the datasets, Droso and Mouse, have a single view of the organism, the ZFish contains two orthogonal, registered but unfused views. To enable treating these views as interchangeable inputs to our networks, we resample them to isotropic resolution.

We use sparse point annotations to train our method. As we leverage annotations originally performed for biological analysis, the annotated lineages are not randomly distributed, instead focusing on the developing nervous system of each organism. Although not necessary for training purposes, annotators ensured that lineages were fully traced by following a cell and all subsequent progeny until they were no longer visible. Thus, there are more annotations in later frames of each recording. See Supplementary Table 1 for the number of cells and divisions annotated in each dataset.

For each organism, we divide the available annotations by time, location, and lineage into train, validation, and test sections, and report results on each split of the data individually, as well as averaged across splits as a form of k-fold cross validation. Cells in *Drosophila* and zebrafish rarely cross the center line of the organism, so we split the lineages into two groups based on side, discarding a small number of zebrafish lineages that did cross the center line. We then train two models using lineages from each side, leaving out 50 central time frames (200-250 for Droso, 150-200 for ZFish) for validation. We test each model on lineages from the side that was not used for training. Supplementary Table 2 shows the number of cells and divisions in each train, validation, and test region for Droso and ZFish.

Within a developing mouse embryo, there is not a clearly defined center line that cells do not cross. Thus, instead of splitting lineages into groups by region, we define three sections of Mouse by time frame: "early" (50-100), "middle" (225-275), and "late" (400-450). Due to extensive embryonic development over the 44 hour recording, early, middle, and late stages represent different cell environments and organization, and there are far more cells by the end of the recording than at the early stages. Each model is trained leaving out two of those sections, one for validation and one for testing. This is repeated for all combinations of validation and testing, resulting in six total train/validation/test splits. The number of cells and division in each Mouse split is shown in Supplementary Table 3.

Supplementary Note 2: Evaluation

Metrics

Cell lineages can be used for a wide variety of analyses, and different kinds of errors can affect downstream results differently; therefore, reducing performance of a tracking method to a single number that represents "overall performance" is generally not possible. Therefore, we distinguish five types of tracking errors: false positive edges (FP), false negative edges (FN), identity switches (IS)—when one reconstructed track takes over following a cell from another reconstructed track—false positive divisions (FP-D) and false negative divisions (FN-D), as shown in Supplementary Figure 1. To allow comparison across datasets, we normalize the number of errors by the number of ground truth edges, resulting in an *errors per edge* metric. Additionally, we compute the fraction of ground truth lineages that were perfectly reconstructed over T time points, for a range of values for T. Ground truth segments over time T were identified using a sliding window of time T over the whole

Dataset	Time (h)	Time Step (min)	Size	Annotated Points	Annotated Divisions
Droso	3.75	0.5	86 GB	75,745	299
Mouse	44.33	5	4.7 TB	37,009	148
ZFish	9.125	1.5	2.1 TB	34,530	88

Supplementary Table 1: Summary information about the three datasets used to develop and evaluate our method.

Split	Train	Validate	Test
Droso side 1	30262 (96)	7327 (54)	38156 (149)
Droso side 2	30688 (107)	7468 (42)	37589 (150)
ZFish side 1	11875 (29)	2121 (6)	14535 (35)
ZFish side 2	12234 (33)	2301 (2)	13996 (35)

Supplementary Table 2: Number of annotated cells (divisions) used for training, validation, and evaluation in Droso and ZFish. Sides of the organisms were arbitrarily labeled 1 and 2, and each split is named for the evaluation side.

Split	Train	Validate	Test
early 1	33522 (132)	3178 (13)	309 (3)
early 2	30287 (109)	6413 (36)	309 (3)
middle 1	33522 (132)	309 (3)	3178 (13)
middle 2	27418 (99)	6413 (36)	3178 (13)
late 1	30287 (109)	309 (3)	6413 (36)
late 2	27418 (99)	3178 (13)	6413 (36)

Supplementary Table 3: Number of annotated cells (divisions) used for training, validation, and evaluation in Mouse. Splits are named for the evaluation set, so early 1 and early 2 are both evaluated on the early section.

evaluation region, splitting at divisions only when they occur in the first frame of the window. Matched reconstructions were considered perfect when none of the error types above occurred over the course of the window.

Evaluating with sparse point annotations presents two unique challenges. First, we cannot determine false positive edges, so we omit this error type from our analysis. Due to the non-maximal suppression window used when extracting cell candidates, our method cannot naively minimize the false negative edge metric by extreme overdetection of false positive cells. However, false positive tracks can still appear, and without dense annotations

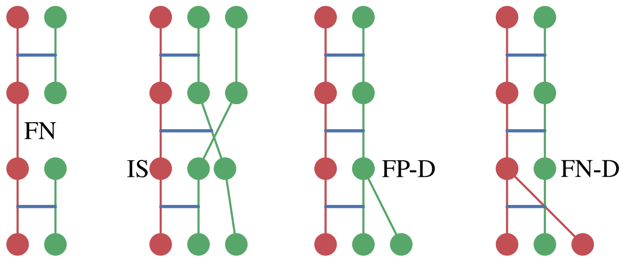

Supplementary Figure 1: Diagram illustrating the four kinds of tracking errors used in our analysis: false negative edges (FN), identity switches (IS), false positive divisions (FP-D) and false negative divisions (FN-D). False positive edges are not pictured, as they cannot be determined from sparse ground truth. Red graphs represent ground truth tracks and green reconstructed tracks, while blue lines represent edges that are matched between the ground truth and reconstructed tracks.

we are limited to qualitative analysis. Second, we cannot use segmentation overlap to match ground truth to reconstructed cells. Instead, we choose a matching threshold that is a bit larger than the radius of a nucleus in the dataset. Considering only nodes within this threshold as potential matching endpoints, we pair ground truth and reconstruction edges using Hungarian Matching to minimize the sum of endpoint distance. Both reconstruction and ground truth edges can be matched to a dummy edge, allowing detection of false negative ground truth edges and reconstructions that do not match to any ground truth.

In addition to evaluating tracking performance, we examine the performance of the cell indicator and movement vector networks. The efficacy of the cell indicator model is determined by the cell recall, or the percent of ground truth cells that have a cell indicator maxima within the matching threshold. To evaluate the quality of the movement vectors, we find the closest cell indicator maxima for each ground truth node (within the matching threshold) and compute the distance between the parent location predicted by the movement vector at that maxima and the actual parent location. We use the ”no movement” prediction as a baseline, to simulate the assumption that cells stay in the same place.

Baselines

Due to the size of our datasets and nature of our ground truth, we can only compare against cell tracking methods that can be run efficiently on multi-terabyte 3D datasets, and that do not require dense annotations or segmentations for training. Tracking with Gaussian Mixture Models (TGMM) (Amat et al., 2014) was previously run on certain time regions of Droso and Mouse, and we were able to extend those results to the full time series. Because TGMM cannot process multi-channel input, for ZFish we produced tracks for each of the two views separately, and reported the best result for each evaluation region. More recently, the tracking method included in the ELEPHANT framework has potential to be scalable to multi-terabyte datsets (Sugawara et al., 2021). The cell detection step requires sparse nuclear segmentations by manual ellipsoid fitting, preventing us from comparing directly with the full method, so instead we run a greedy nearest-neighbor linking algorithm inspired by this work on our cell candidates. Starting in the final frame t, we consider all cell candidates to be part of a track. We then greedily select edges from t to $t-1$ with the smallest difference between predicted and actual offset, enforcing the constraint that cells cannot divide into more than two by removing edges that connect to nodes in $t-1$ that already have two selected incoming edges. We then process each subsequent pair of frames going back in time, first extending existing tracks, and then creating new tracks if any valid edges remain.

Results

Figure 2 shows the sum of errors per edge for each organism, averaged over the train/validation/test splits. Across all datasets, our method produces significantly fewer errors per edge than TGMM

and the greedy baseline, with the greedy baseline landing between TGMM and our method. Our method performs similarly between Mouse and Droso (0.049 and 0.050 total errors per edge) and slightly worse on ZFish (0.078).

Considering individual types of errors provides more insight into the performance of the different methods. For both TGMM and our method, false negative edges (FN) are the most common error type. In every case, our method produces fewer FN and fewer false positive divisions (FP-D) than TGMM. The false negative division (FN-D) performance is similar between our method and TGMM - in absolute numbers, neither our method nor TGMM correctly identifies more than a third of the divisions, but divisions are so underrepresented in the evaluation sets that this error type does not significantly affect the overall sum of errors. On Mouse, our method does not produce any divisions, and thus the FP-D rate is always zero, while TGMM has a very high FP-D rate and still does not detect many of the true divisions.

While our method does not always have fewer identity switches (IS) than TGMM, exmaning performance by dataset shows clear trends. For Mouse, our method always produces fewer IS than TGMM. However, for Droso and ZFish, TGMM produces hardly any IS, likely due to the high number of FN. Since an IS can only occur when two neighboring ground truth edges are matched to different reconstructed tracks, a high number of FN reduces the opportunities for IS to occur. Our method significantly reduces the number of FN on these datasets, resulting in slightly more IS than TGMM but fewer overall errors.

Figure 2 also shows the track accuracy, or fraction of perfectly reconstructed tracks, for a range of track lengths. For length 1, this metric is the fraction of false negative edges, and thus our method and the greedy baseline outperform TGMM. However, as the time window increases, the greedy baseline accuracy drops quickly for all datasets, reflecting the lack of global track optimization in this per-frame tracking algorithm. TGMM's accuracy is similar to the greedy baseline on Mouse, but for Droso and ZFish, the slope of the decline is much flatter, indicating that TGMM perfectly reconstructs more long track segments on these datasets. Combining the fairly high track accuracy of TGMM with the large number of false negative edges, we can infer that on Droso and ZFish, TGMM's errors are grouped together, resulting in some tracks being faithfully reconstructed and others missed completely. Our method has the highest track accuracy across all datasets, with a similar rate of decline as TGMM on Droso and ZFish but a higher starting accuracy.

We further investigate which portion of the errors stem from inaccurate cell detections versus errors introduced during the discrete optimization due to suboptimal node and edge scores. To this end, we computed a *best effort* solution, *i.e.*, the best solution the discrete solver could obtain with the given candidate graph. In Supplementary Figure 4 we contrast the fraction of correctly reconstructed segments over t time frames of our solution with the best effort. With the exception of the zebrafish dataset (which has lower cell recall, see Supplementary Figure 5), we find that the majority of tracking errors could be avoided by providing better scores to the discrete solver (cell indicator scores and movement vector estimates).

To examine differences in performance between models trained and evaluated on tracks from different regions, we show results for each train/test split described in Supplementary Note 1 in Supplementary Figure 2 (errors per edge) and Supplementary Figure 3 (track accuracy). Due to the cross validation used for Mouse, we have results from two models for each evaluation region, with each model trained and validated on different data splits. For both sum of errors and track accuracy, the models that were trained on more data (early 1, middle 1, and late 1 as shown in Supplementary Table 3) performed slightly better than those trained on less. The sum of errors also slightly increased for our method from early to late regions on Mouse, reflecting increasing difficulty of the task over time, although overall trends about relative performance and error types between our method and the baselines hold. Droso shows similar results between the two evaluation regions, but the same is not true for ZFish. TGMM performs much better on ZFish side 2 than side 1 in both track accuracy and sum of errors. Indeed, on side 2, TGMM and our method have a similar track accuracy, while TGMM performance on side 1 degrades significantly using both metrics. Manual examination of the raw data shows that the tracks on side 1 are harder for a human to identify due to less clear signal on that side of the dataset; thus, the relative results indicate that our method is more robust to varied imaging conditions than TGMM. Unexpectedly, the greedy baseline performs worse on the easier side 2. To explain this, we observe that the cell indicator model for side 2 predicts significantly more candidate cells, and more false positive candidates, than the model for side 1, likely due to randomness in the training pipeline (see Supplementary Note 3). The greedy method creates many false positive divisions involving those candidates, while ours does not, showing that the optimization step can extract coherent tracks from a noisy candidate graph.

Supplementary Figure 5 shows the standalone performance of the cell indicator and movement vector networks. Cell recall for all mouse and *Drosophila* models exceeds 0.99, indicating that nearly all ground truth cells in these datasets have a nearby candidate cell. Recall is slightly lower for both zebrafish models, but still exceeds 0.96. The movement vector network has a smaller mean distance between predicted and actual parent location than the baseline for all models, and the distribution of distances is concentrated closer to zero. The mouse cells move further on average than the *Drosophila* and zebrafish cells, so the magnitude of improvement compared for mouse is greater. The max distance is higher for our model than the baseline in all but one case, indicating that in the scenarios where cells move the most, such as after division, the movement vector network can point the wrong way. However, overall the movement vector network tends to point in the direction of the parent, as expected.

The sparse ground truth annotations used so far do not allow us to evaluate false positive detections. To count false positive detections, we manually picked three regions in the Mouse dataset (in the "early", "middle", and "late" parts of the dataset), each with a side length of 80μm, spanning 10 frames. The regions were chosen to contain cells with low contrast that are still possible for humans to follow. The selection of the regions was blind to our automatic reconstruction. We then manually evaluated the automatic reconstruction in those regions, labelling false positives and identity switches. While we found identity switches in those regions (9, 5, and 2, respectively for "early", "middle", and "late"), we did not observe false positive cell detections, suggesting that the ILP is successfully in filtering non-contiguous false positive cell detections.

Cell Tracking Challenge

We further evaluated our method through a submission to the Cell Tracking Challenge[1] (Ulman et al., 2017). Specifically, we submitted results for the two 3D+t datasets Fluo-N3DH-CE and Fluo-N3DL-DRO, for which we used only the training data provided by the challenge organizers. For the Fluo-N3DH-CE dataset, a developing *C. Elegans* embryo was imaged with a voxel size of $0.09 \times 0.09 \times 1$µm at a temporal resolution of 1.5 minutes using a Zeiss LSM 510 Meta (Murray et al., 2008). The Fluo-N3DL-DRO dataset shows a developing *Drosophila* embryo imaged with a voxel size of 0.406×0.406×2.03µm at a temporal resolution of 30 seconds using a SIMView light-sheet microscope (Amat et al., 2014). On both datasets, our method (named Jan-US on the challenge website) led, at the time of submission to the challenge, the board in terms of the Tra score (a score to measure tracking accuracy, with a score of 1 being perfect, see Ulman et al. (2017) for details) and on Fluo-N3DH-CE additionally in terms of the Det score (an analogous score to measure detection accuracy). On Fluo-N3DH-CE we additionally incorporate a cell state classifier to improve the performance on divisions (Hirsch et al., 2022): We observed a modest improvement with a Tra score of 0.979 compared to the previously best score of 0.975 out of 13 submissions and a Det score of 0.981 compared to the previously best score of 0.979 out of 18 submissions; this has since been surpassed by a new submission (Tra 0.987, Det 0.990). On the more challenging Fluo-N3DL-DRO dataset, we reach a Tra of 0.785, thus substantially improving over the previously best score of 0.668 out of five submissions.

Supplementary Note 3: Ablation Study

Our training method contains multiple sources of randomness, from batch sampling to augmentation. To determine the effect of this randomness, we train, validate, and test the same model five times. The results shown in Supplementary Figure 6a illustrate that random batch selection and augmentation in training do affect tracking performance. The sum of errors and distribution of errors between false negative edges and identity switches vary substantially between the five models.

In addition to our standard model, we test three changes to architecture, sampling, and augmentation. In our U-Net architecture, we experiment with two different upsampling methods: with and without limiting the transpose convolutional kernel to a kernel of ones. We call these two upsampling methods transpose upsampling (TU) and constant upsampling (CU). Because divisions are underrepresented in the training data and particularly difficult, we try sampling batches specifically at divisions 25% of the time (+D). We also simulate more cell movement by adding a random shift augmentation between the previous frame and the target frame (+S).

Results for each combination of these training and architecture variations on one Mouse split are shown in Supplementary Figure 6b. To draw conclusions about the effect of any of these variations, the resulting change in performance has to be greater than the effect of random retraining shown in Supplementary Figure 6a. In general, none of the models produced a large, consistent difference in tracking score, although division sampling seems to produce worse results in general. Due to training time and expense, we were not able to train every model in the ablation study multiple times or on every dataset, which would have allowed more conclusive comparisons. While further exploration into architecture and training decisions could yield incremental improvements, these initial results are insufficient to incorporate any of the three changes into our main model.

1. http://celltrackingchallenge.net

	FN	IS	FP-D	FN-D	Sum
block-wise	1154	656	71	114	1995
global	1122	633	122	96	1973

Supplementary Table 4: Error comparison between block-wise and global ILP inference on the Droso dataset.

	FN	IS	FP-D	FN-D	Sum
GT l and m	0	0	0	0	0
l ± 5µm	0	0	0	0	0
l ± 10µm	4	2	0	0	6
l ± 15µm	657	2	4	5	668
m ± 5µm	2	0	0	0	2
m ± 10µm	0	2	1	0	3
m ± 15µm	2	0	1	0	3
m ± 20µm	28	6	5	0	39
m ± 25µm	74	2	6	2	84
m ± 30µm	417	6	0	13	436
predicted l and m	94	34	0	13	141

Supplementary Table 5: Errors for different random perturbations of ground truth location l and movement vectors m on Mouse (middle).

We further investigated the effect of the block-wise solving of the tracking ILP, compared to a globally optimal solution over full frames on the Droso dataset (split Droso side 2, the maximum block size is restricted by the size of the validation set, in this case 50 frames per block with a total of 450 frames). A detailed summary of error types is given in Supplementary Table 4. In total, the block-wise inference results in 1.1% more errors compared to the global solution in the same time interval.

Similarly, we investigated the sensitivity of ILP inference to imprecise predictions. For that, we used the available ground truth as a drop-in replacement for actual detections, and computed the number of errors made for different amounts of noise added (uniformly distributed within different intervals). Results are shown in Supplementary Table 5. Generally, the ILP solution is robust to perturbations up to 10µm for cell locations and 15µm for movement vectors. Larger perturbations lead primarily to FNs, since the cells are moved further than one cell radius (≈ 10µm) and are no longer counted as a TP.

Finally, we investigated the impact of the movement vectors on the performance on the Droso dataset (split Droso side 2). Solving the ILP without them results in a ≈ 5% increase in errors and the number of correctly reconstructed divisions drops to almost zero (≈ 1% vs ≈ 25-30% of divisions correctly reconstructed).

Supplementary Note 4: Amount of Training Data

Contemporary machine learning methods require substantial amounts of training data to provide accurate predictions. To study the relationship between the amount of annotated cells (including links over time and divisions), we retrained our models using varying amounts of the available ground truth on the Droso and Mouse datasets (see Supplementary Table 1 for the total amount of annotations per sample, and Supplementary Table 2 and Supplementary Table 3 for the amount used for training and validation for Droso and Mouse, respectively).

Supplementary Figure 7a shows the progression of the number of errors for 1%, 5%, 10%, 20%, 40%, 60%, 80%, and 100% of the ground truth used on the Droso dataset (split Droso side 2). Reported numbers are averages over three independent training and evaluation runs to account for variations due to random subsampling of the training data and initialization of the network. On this dataset, we observe that training on more than 40% of the available training data (corresponding to $\approx$ 12000 annotated cells and $\approx$ 50 divisions) does not lead to noticeable improvements.

We observe a similar effect on the Mouse dataset (split Mouse late 2), shown in Supplementary Figure 7b. On this dataset, we see a similar saturation after 40% (corresponding to $\approx$ 11000 annotated cells and $\approx$ 40 divisions).

Those results suggest that incremental generation of training data might be a viable strategy to minimize the amount of total annotations needed.

Supplementary Discussion

Using deep learning allows the method to adapt to different imaging conditions and organisms, and boosts performance compared to a heuristic approach as shown by the comparative performance of TGMM and the greedy baseline. However, deep learning in general requires annotated training data, which can be time consuming and costly to acquire. Our method minimizes the annotation burden by leveraging sparse point annotations in segments as short as two frames. Furthermore, the amount of training data required is reduced because the models do not need to achieve perfect performance: the global optimization can filter out superfluous detections and ignore individual inaccuracies in favor of global evidence and biological priors. Based on our results, between 10 and 30 thousand sparse point annotations created with MaMuT or Masodon would be sufficient to train a model to track cells in new organisms or imaging conditions. Assuming each point annotation can be generated in 3 seconds, sufficient training data can be produced in 8 to 24 hours of manual annotation.

The unavoidable uncertainty in the placed annotations with respect to the exact correct location is one reason for the use of Gaussians. Another is to facilitate smooth transitions in the gradient-based learning process. Yet, particularly as we are not interested in segmentation, the Gaussians do not have to match the shape of the corresponding cell precisely. They have to be sufficiently small to always allow for a distinction of neighbouring cells and large enough to provide a stable training signal. The method is quite robust with respect to the variability in shape and size of the cells. This can be attested by, for instance, its ability to correctly handle the elongated cells in the Droso dataset. However, there are limits. In case of severely varying sizes, as during the development of *C. elegans* embryos, the width of the Gaussians has to be adjusted.

While the optimization step filters out some false positive candidate detections in the backround, even better performance could be achieved by including negative examples in training. While we cannot quantitatively measure false positives when evaluating with sparse annotations, qualitative analysis of the results showed that the cell indicator network does predict false positives, especially in regions with high intensity but no discernible nuclei. When these false positive candidates persist through multiple frames, they can be linked to create false positive tracks. Incorporating our cell indicator model into the ELEPHANT interactive training and annotation framework (Sugawara et al., 2021) is a possible solution, since annotators could easily generate negative training examples in regions where the network most needs guidance. Using targeted negative examples, we expect the cell indicator network could learn to suppress cell prediction in these high intensity regions with limited fine-tuning.

Conversely, false negative detections are detrimental to our method. Although we have not observed this to be a problem on the datasets investigated in this study, false positives have the potential to disrupt the lineage tracking. The discrete optimization performed via the ILP can only remove candidate nodes and edges, but does currently not add missing ones. This might pose a problem for datasets where cells or their nuclei are sporadically invisible.

When applying our method to a new dataset, one bottleneck of the method is the need to grid search the hyperparameters of the ILP. Even with blockwise processing, solving the ILP on the whole validation set takes tens of minutes per run, so we limited the grid search to four values per hyperparameter, resulting in 256 runs. We were guided by experience in choosing the range of values to search, but there is no guarantee that our solution was optimal, or that the same range would apply to different datasets. In the future, we will examine alternatives to grid searching a manually selected range of values, such as using a structured support vector machine to find the best set of ILP parameters on a given dataset.

Finally, identifying divisions is an important question for developmental analysis, but divisions are underrepresented compared to non-dividing cells, and have distinct movement and appearance. To address the difficulties that divisions present, we tried sampling divisions more frequently during training and adding a shift augmentation to mimic the movement of dividing cells, as discussed in Supplementary Note 3. However, even with these tactics, our method does not consistently identify divisions. One possible explanation is that the failure to identify divisions occurs in the optimization step, while our interventions focus on improving network predictions. During validation, we choose the ILP hyperparameters that minimize the sum of all error types. The underrepresentation of divisions in the validation set means that false negative divisions do not contribute heavily to the sum compared to false negative edges or even false positive divisions. Minimizing sum of errors thus can lead to models that select very few divisions, as long as the other error categories are minimized. Focused efforts to improve division performance will be necessary to attain reliable results.

Acknowledgements

We thank William Patton and Tri Nguyen for supporting the Gunpowder and Daisy libraries, Steffen Wolf for his guidance and feedback, and Nils Eckstein, Julia Buhmann, and Arlo Sheridan for helpful discussions. **Funding** This work was supported by Howard Hughes Medical Institute. K.D. was supported by the Medical Research Council, as part of United Kingdom Research and Innovation [MCUP1201/23]. P.H. was supported by HFSP grant RGP0021/2018-102, the MDC Berlin-New York University exchange program. P.H. and D.K. were supported by the HHMI Janelia Visiting Scientist Program. **Author contributions** *Conceptualization*: Jan Funke, Philipp J. Keller. *Funding acquisition*: Jan Funke, Philipp J. Keller, Stephan Preibisch, Katie McDole, Peter Hirsch, Dagmar Kainmueller. *Software*: Caroline Malin-Mayor, Peter Hirsch, Jan Funke, Leo Guignard. *Validation and evaluation*: Caroline Malin-Mayor, Peter Hirsch *Data and annotation*

generation: Katie McDole, Yinan Wan, William C. Lemon. *Supervision*: Jan Funke, Stephan Preibisch, Philipp J. Keller, Dagmar Kainmueller. *Writing - original draft*: Caroline Malin-Mayor, Jan Funke. *Writing - review & editing*: Caroline Malin-Mayor, Jan Funke, Philipp J. Keller, Katie McDole, Stephan Preibisch, Peter Hirsch, Leo Guignard, Dagmar Kainmueller.

Data Availability

Raw datasets are available through their respective publications (Mouse: McDole et al. (2018), Droso: Amat et al. (2014), ZFish: Wan et al. (2019)).

Ground-truth annotations used for training and evaluation of our method, together with the lineage tracks produced by our method, are publicly available at https://github.com/funkelab/linajea_experiments.

Figure 1:	Mouse: raw data, training data, reconstructions
Figure 2:	Mouse, Droso, ZFish: raw data, ground-truth annotations, reconstructions
Sup. Figure 2:	Mouse, Droso, ZFish: ground-truth annotations, reconstructions
Sup. Figure 3:	Mouse, Droso, ZFish: ground-truth annotations, reconstructions
Sup. Figure 4:	Mouse, Droso, ZFish: ground-truth annotations, reconstructions
Sup. Figure 5:	Mouse, Droso, ZFish: ground-truth annotations, reconstructions
Sup. Figure 6:	Mouse: ground-truth annotations, reconstructions
Sup. Figure 7	Mouse, Droso: ground-truth annotations, reconstructions

Code Availability

The code used to train networks, reconstruct lineages, and evaluate the results is available in the "linajea" repository, https://github.com/funkelab/linajea.

References

Amat, F., Lemon, W., Mossing, D. P., McDole, K., Wan, Y., Branson, K., Myers, E. W., and Keller, P. J. (2014). Fast, accurate reconstruction of cell lineages from large-scale fluorescence microscopy data. *Nature Methods*, 11(9):951–958.

Hirsch, P., Malin-Mayor, C., Preibisch, S., Kainmueller, D., and Funke, J. (2022). Tracking by weakly-supervised learning and graph optimization for whole-embryo c. elegans lineages. *arXiv*.

McDole, K., Guignard, L., Amat, F., Berger, A., Malandain, G., Royer, L. A., Turaga, S. C., Branson, K., and Keller, P. J. (2018). In Toto Imaging and Reconstruction of Post-Implantation Mouse Development at the Single-Cell Level. *Cell*, 175(3):859–876.e33.

Murray, J. I., Bao, Z., Boyle, T. J., Boeck, M. E., Mericle, B. L., Nicholas, T. J., Zhao, Z., Sandel, M. J., and Waterston, R. H. (2008). Automated analysis of embryonic gene expression with cellular resolution in C. elegans. *Nature Methods*, 5(8):703–709.

Sugawara, K., Cevrim, C., and Averof, M. (2021). Tracking cell lineages in 3D by incremental deep learning. *bioRxiv*, page 2021.02.26.432552.

Ulman, V., Maška, M., Magnusson, K. E. G., Ronneberger, O., Haubold, C., Harder, N., Matula, P., Matula, P., Svoboda, D., Radojevic, M., Smal, I., Rohr, K., Jaldén, J., Blau, H. M., Dzyubachyk, O., Lelieveldt, B., Xiao, P., Li, Y., Cho, S.-Y., Dufour, A. C., Olivo-Marin, J.-C., Reyes-Aldasoro, C. C., Solis-Lemus, J. A., Bensch, R., Brox, T., Stegmaier, J., Mikut, R., Wolf, S., Hamprecht, F. A., Esteves, T., Quelhas, P., Demirel, Ö., Malmström, L., Jug, F., Tomancak, P., Meijering, E., Muñoz-Barrutia, A., Kozubek, M., and Ortiz-de Solorzano, C. (2017). An objective comparison of cell-tracking algorithms. *Nature Methods*, 14(12):1141–1152.

Wan, Y., Wei, Z., Looger, L. L., Koyama, M., Druckmann, S., and Keller, P. J. (2019). Single-Cell Reconstruction of Emerging Population Activity in an Entire Developing Circuit. *Cell*, 179(2):355–372.e23.

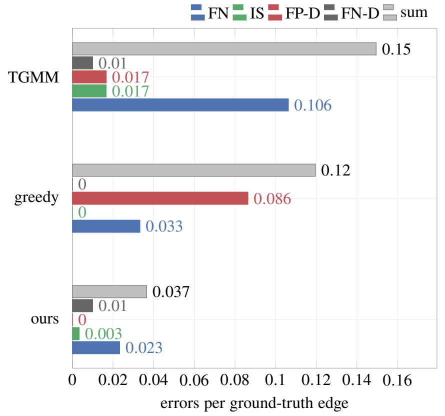

(a) Mouse early 1

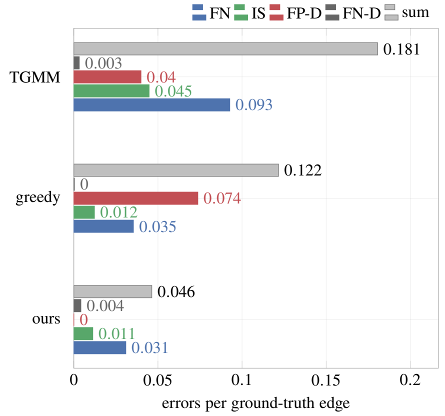

(b) Mouse middle 1

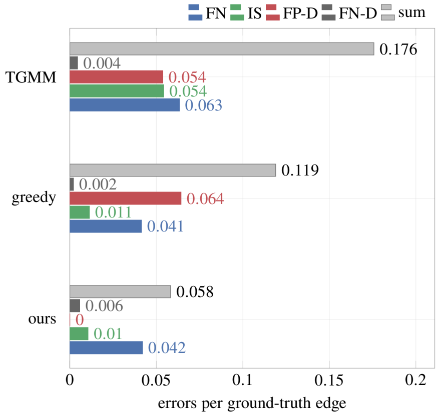

(c) Mouse late 1

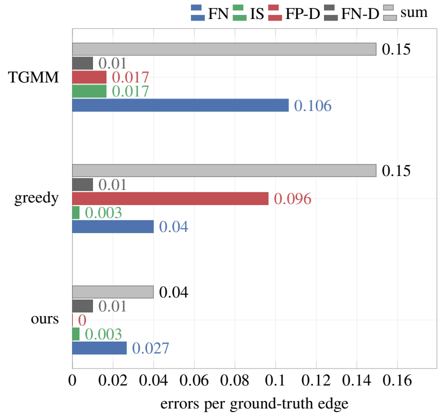

(d) Mouse early 2

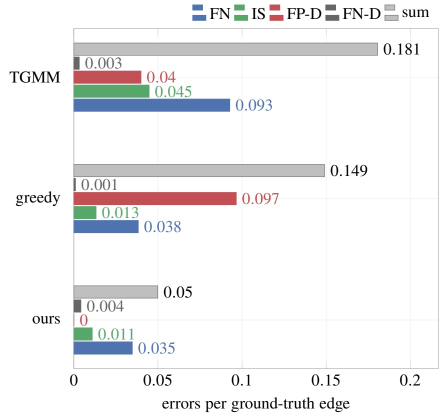

(e) Mouse middle 2

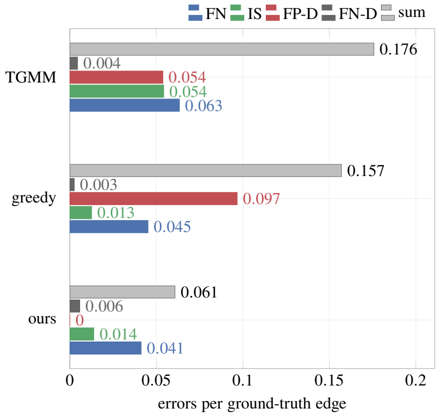

(f) Mouse late 2

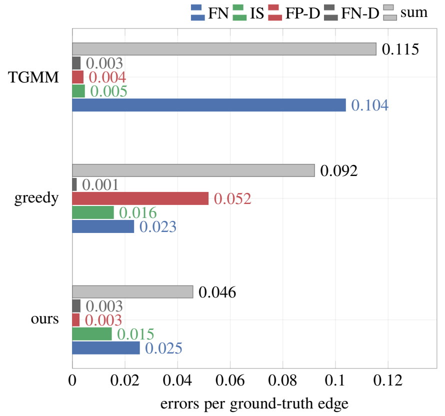

(g) Droso side 1

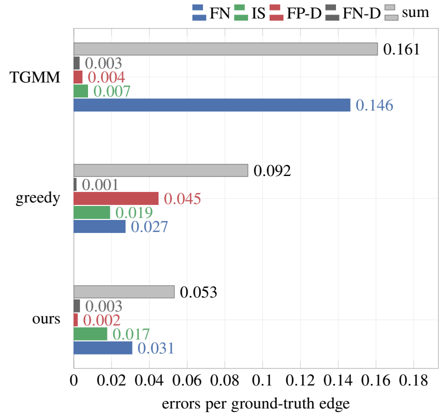

(h) Droso side 2

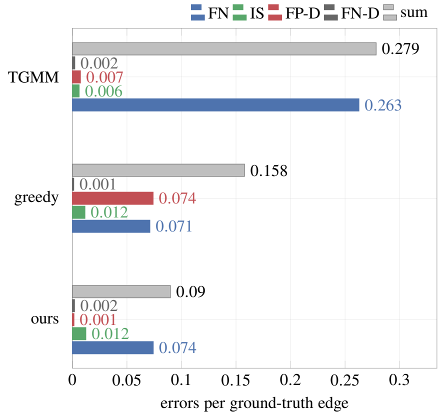

(i) ZFish side 1

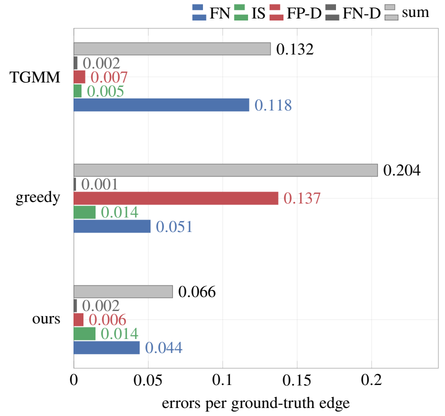

(j) ZFish side 2

Supplementary Figure 2: Errors per edge for our method, the greedy baseline, and TGMM, on all datasets and evaluation regions.

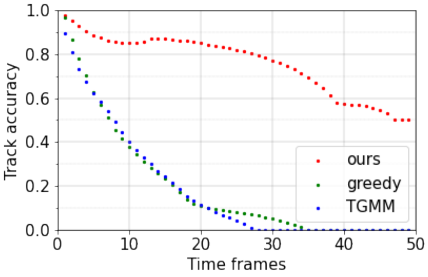

(a) MOUSE early 1

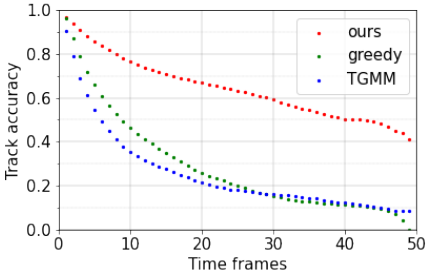

(b) MOUSE middle 1

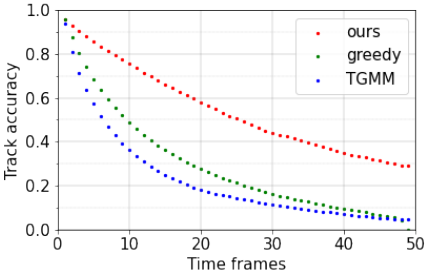

(c) MOUSE late 1

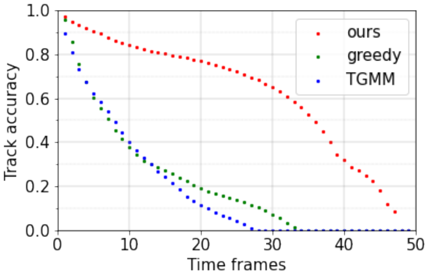

(d) MOUSE early 2

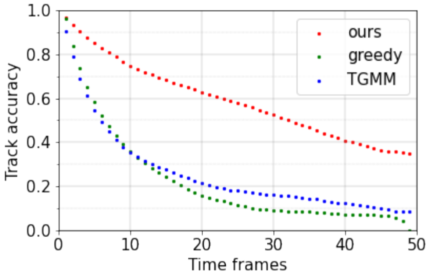

(e) MOUSE middle 2

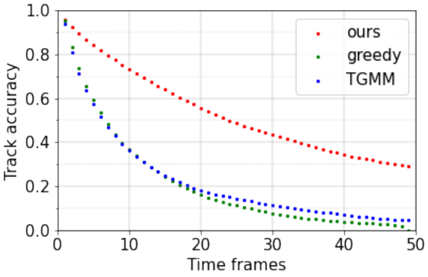

(f) MOUSE late 2

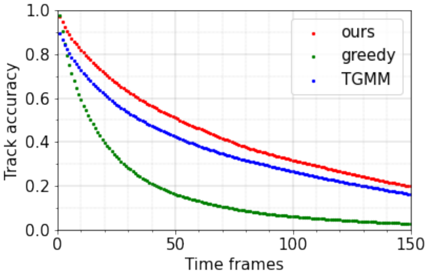

(g) DROSO side 1

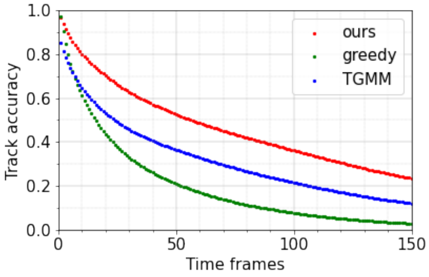

(h) DROSO side 2

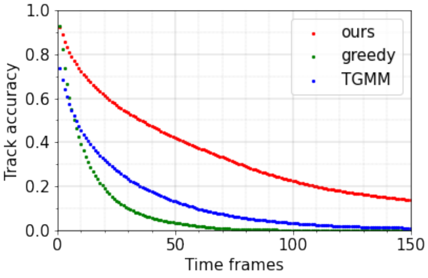

(i) ZFISH side 1

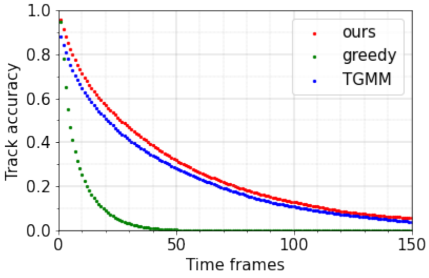

(j) ZFISH side 2

Supplementary Figure 3: Fraction of ground truth segments correctly reconstructed over t time frames, for a range of t, on all datasets and evaluation regions.

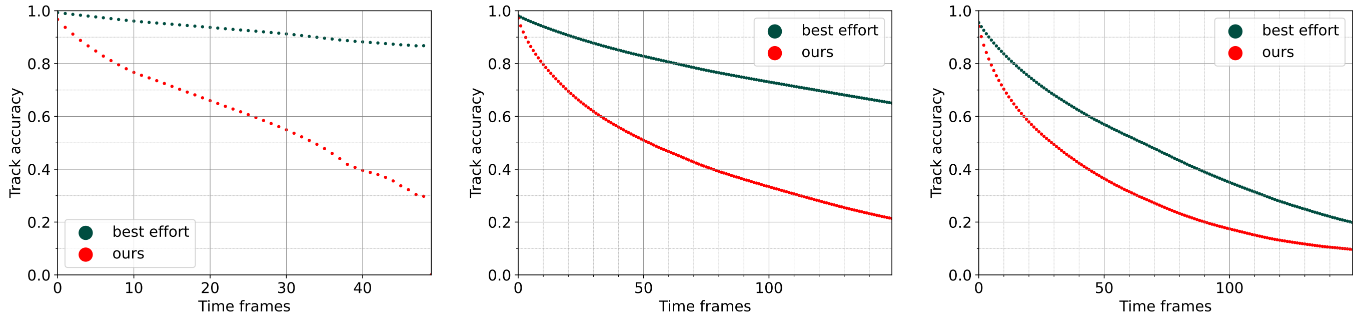

(a) Mouse (b) Droso (c) ZFish

Supplementary Figure 4: Fraction of ground truth segments correctly reconstructed over t time frames, for a range of t. *ours* is identical to Figure 2b, *best effort* shows the best solution attainable by the discrete solver, given the found cell candidates and edges between frames.

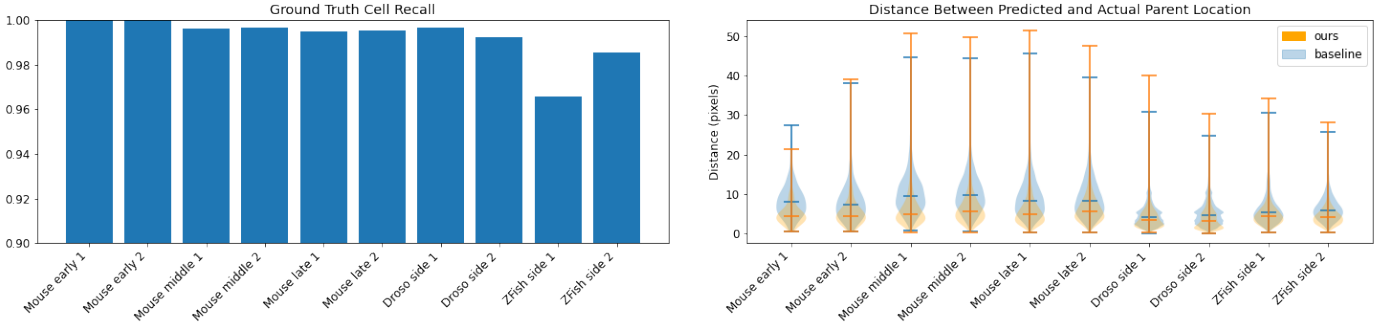

(a) cell indicator network (b) movement vector network

Supplementary Figure 5: Performance of the cell indicator and movement vector networks. **(a)** Recall of cell indicator networks, as measured by the number of ground truth annotations in the evaluation set that have a cell indicator maxima within the matching threshold. **(b)** The distance between the predicted parent locations and actual parent locations for each ground truth cell with a matched candidate within the matching threshold, represented as a violin plot with hashes at the min, max and median values. Baseline of no movement is shown in blue, and our predicted movement vectors are shown in orange.

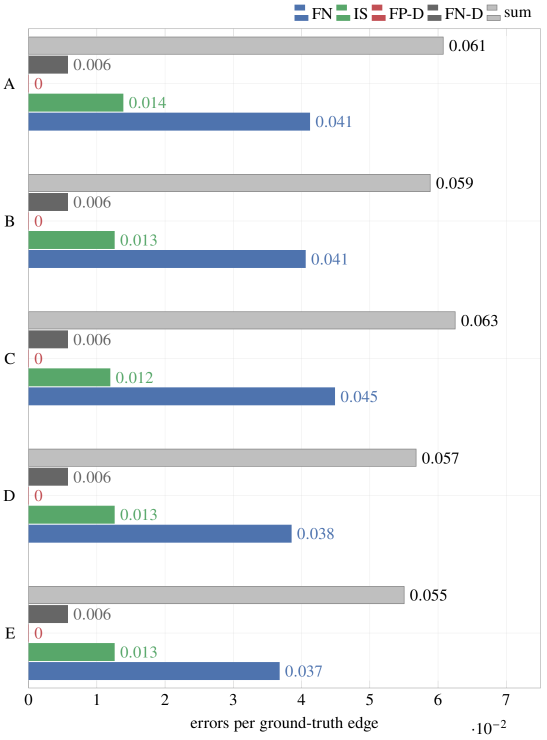

(a) Effect of Retraining

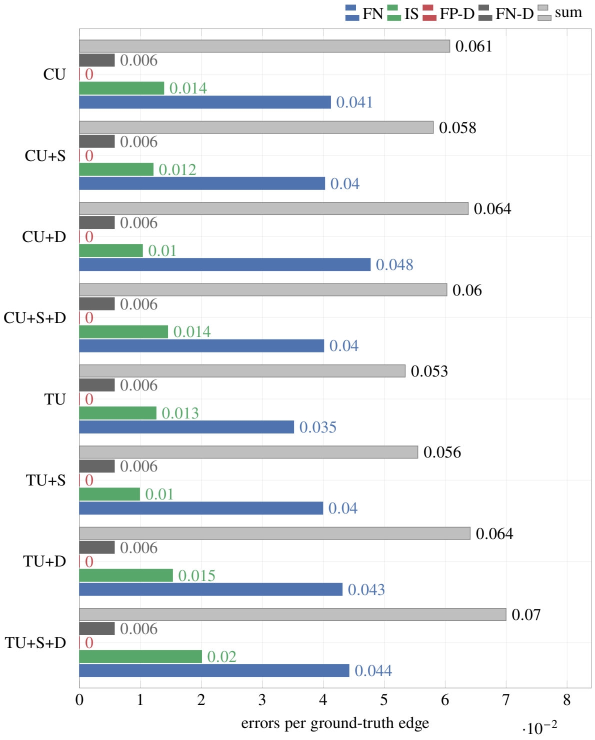

(b) Architecture and Training Variations

Supplementary Figure 6: Supplementary experiments to determine the effect of randomness in training and architecture and training variations. **(a)** Sum of errors per ground truth edge, for five copies of the same model trained, validated, and tested on Mouse late 2. Variation in the error counts stems from random network initialization and random training sample selection and augmentation. **(b)** Sum of errors per ground truth edge for eight different models trained, validated, and tested on Mouse late 2, comparing constant (CU) and transpose upsampling (TU), shift augmentation (+S), and division sampling (+D).

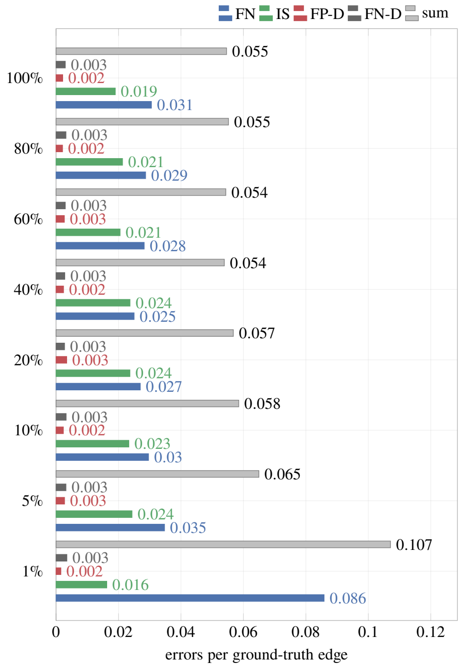

(a) Droso side 2

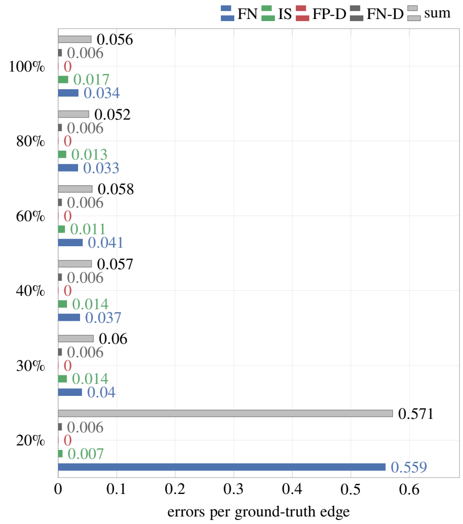

(b) Mouse late 2

Supplementary Figure 7: Reconstruction errors for different amounts of training data on Droso and Mouse
