# Supplementary material for: Automated reconstruction of whole-embryo cell lineages by learning from sparse annotations
Source: Nat Biotechnol. 2022 Sep 5;41(1):44–9. doi: 10.1038/s41587-022-01427-7 (PMC7614077; doi:10.1038/s41587-022-01427-7)
Supplement: Supplementary file 2 — Reporting Summary [file 41587_2022_1427_MOESM2_ESM.pdf]

Corresponding author(s): Jan Funke

Last updated by author(s): 2022/06/28

Reporting Summary

Nature Research wishes to improve the reproducibility of the work that we publish. This form provides structure for consistency and transparency in reporting. For further information on Nature Research policies, see our Editorial Policies and the Editorial Policy Checklist.

Statistics

For all statistical analyses, confirm that the following items are present in the figure legend, table legend, main text, or Methods section.

n/a	Confirmed	
☐	☒	The exact sample size (*n*) for each experimental group/condition, given as a discrete number and unit of measurement
☐	☒	A statement on whether measurements were taken from distinct samples or whether the same sample was measured repeatedly
☒	☐	The statistical test(s) used AND whether they are one- or two-sided<br>*Only common tests should be described solely by name; describe more complex techniques in the Methods section.*
☒	☐	A description of all covariates tested
☒	☐	A description of any assumptions or corrections, such as tests of normality and adjustment for multiple comparisons
☒	☐	A full description of the statistical parameters including central tendency (e.g. means) or other basic estimates (e.g. regression coefficient) AND variation (e.g. standard deviation) or associated estimates of uncertainty (e.g. confidence intervals)
☒	☐	For null hypothesis testing, the test statistic (e.g. *F*, *t*, *r*) with confidence intervals, effect sizes, degrees of freedom and *P* value noted<br>*Give P values as exact values whenever suitable.*
☒	☐	For Bayesian analysis, information on the choice of priors and Markov chain Monte Carlo settings
☒	☐	For hierarchical and complex designs, identification of the appropriate level for tests and full reporting of outcomes
☒	☐	Estimates of effect sizes (e.g. Cohen's *d*, Pearson's *r*), indicating how they were calculated

Our web collection on statistics for biologists contains articles on many of the points above.

Software and code

Policy information about availability of computer code

Data collection	Standard python packages (networkx, pymongo, scipy, pandas, tensorflow, zarr, sklearn, gunpowder, daisy), see https://github.com/funkelab/linajea for a full list of requirements and https://github.com/funkelab/linajea_experiments for training and inference code.
Data analysis	Standard python packages (pymongo, scipy, pandas, zarr, sklearn), see https://github.com/funkelab/linajea for a full list of requirements and https://github.com/funkelab/linajea_experiments for evaluation code.

For manuscripts utilizing custom algorithms or software that are central to the research but not yet described in published literature, software must be made available to editors and reviewers. We strongly encourage code deposition in a community repository (e.g. GitHub). See the Nature Research guidelines for submitting code & software for further information.

Data

Policy information about availability of data

All manuscripts must include a data availability statement. This statement should provide the following information, where applicable:

- Accession codes, unique identifiers, or web links for publicly available datasets
- A list of figures that have associated raw data
- A description of any restrictions on data availability

Raw datasets are available through their respective publications (Mouse: McDole et al. (2018), Droso: Amat et al. (2014), ZFish: Wan et al. (2019b)). Ground-truth annotations used for training and evaluation of our method, together with the lineage tracks produced by our method, are publicly available at https://github.com/funkelab/linajea_experiments.

Field-specific reporting

Please select the one below that is the best fit for your research. If you are not sure, read the appropriate sections before making your selection.

☒ Life sciences ☐ Behavioural & social sciences ☐ Ecological, evolutionary & environmental sciences

For a reference copy of the document with all sections, see nature.com/documents/nr-reporting-summary-flat.pdf

Life sciences study design

All studies must disclose on these points even when the disclosure is negative.

Sample size	The number of annotated cells (with links to previous frames) used for training and evaluation is given by the available ground-truth, i.e., 75,745 for the Drosophila dataset, 37,009 for the Mouse dataset, 34,530 for the Zebrafish dataset.
Data exclusions	No data was excluded.
Replication	Results can be replicated. The effect of random initialization of networks for training is reported in Supplementary Figure 6a.
Randomization	N/A
Blinding	N/A

Reporting for specific materials, systems and methods

We require information from authors about some types of materials, experimental systems and methods used in many studies. Here, indicate whether each material, system or method listed is relevant to your study. If you are not sure if a list item applies to your research, read the appropriate section before selecting a response.

Materials & experimental systems

n/a	Involved in the study
☒	☐ Antibodies
☒	☐ Eukaryotic cell lines
☒	☐ Palaeontology and archaeology
☒	☐ Animals and other organisms
☒	☐ Human research participants
☒	☐ Clinical data
☒	☐ Dual use research of concern

Methods

n/a	Involved in the study
☒	☐ ChIP-seq
☒	☐ Flow cytometry
☒	☐ MRI-based neuroimaging
